# Topical gabapentin and its relation to cutaneous innervation in symptomatic lymphocytic primary cicatricial alopecia

**DOI:** 10.1002/ski2.381

**Published:** 2024-04-01

**Authors:** Sara Bohjanen, Brian D. McAdams, Nasia Mead, Adam Loavenbruck, George L. Wilcox, Briana Paiewonsky, Javed Shaik, Maria K. Hordinsky

**Affiliations:** ^1^ Department of Dermatology University of Minnesota Minneapolis Minnesota USA; ^2^ Department of Neurology University of Minnesota Minneapolis Minnesota USA; ^3^ Department of Neuroscience University of Minnesota Minneapolis Minnesota USA

## Abstract

In this pilot study, participants with symptomatic lymphocytic primary cicatricial alopecia applied 6% topical gabapentin solution twice daily to affected areas for 12 weeks. There was a significant reduction in symptoms, but no pronounced effect on nerve fibre density or neuropeptide expression.

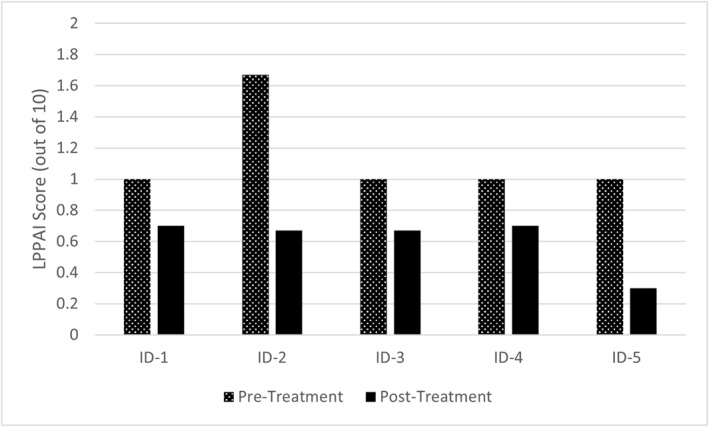

Dear Editor,

Gabapentin is a neuromodulator used to treat neuropathic pain and different forms of chronic pruritus.^1^ In this pilot study, we aimed to examine the effect of 6% topical gabapentin solution on the scalp‐related symptoms and signs of participants with lymphocytic primary cicatricial alopecia (PCA). Decreased epidermal nerve fibre density (ENFD) is associated with neuropathies, and recent evidence in a murine model of neuropathy suggests that gabapentin modifies cutaneous nerve density.^2^ We hypothesised that by influencing cutaneous innervation, topical gabapentin would alleviate the pain, itch or burning sensations that patients with symptomatic lymphocytic PCA experience.

In this pilot study (NCT03346668), participants with lymphocytic PCA experiencing scalp‐related itching, pain, and burning were instructed to apply 1 mL of topical 6% gabapentin solution to affected areas twice daily for 12 weeks. This dose was previously used in a study of topical gabapentin for the treatment of vulvodynia, where improvement of pain was reported after 8 weeks of topical gabapentin use.^3^ The formulation used in our study consisted of 6 g gabapentin powder, 0.16 g sodium phosphate monobasic anhydrous powder, 0.758 g sodium phosphate dibasic anhydrous powder, and 100 mL sterile water. No other treatment was allowed. During monthly visits, participants completed questionnaires and the principal investigator assessed disease severity by using the Lichen Planopilaris Activity Index (LPPAI)^4^ for all participants. Side effects of topical gabapentin and gabapentin blood levels were also monitored. Punch biopsies (4‐mm) of affected and unaffected scalp were collected at baseline (visit 1) and at 12 weeks (visit 5). Specimens were processed and ENFD, substance P (SP), and calcitonin gene‐related peptide (CGRP) were quantified as described by Doche et al.^5^ The study had Institutional Review Board approval (1308M40801), and participants provided written, informed consent prior to enrolment.

Participants included three subjects with symptomatic LPP, one with frontal fibrosing alopecia (FFA), and one with central centrifugal cicatricial alopecia (CCCA). There were three female and two male participants. The average age was 52 years, with a range of 37–75 years. Three participants had previously been treated with both topical and intralesional steroids and one with hydroxychloroquine. Most participants (4 of 5) completed the study through visit 6, but one participant voluntarily withdrew from the study at visit 5 due to lack of perceived efficacy. Blood gabapentin levels were negligible for all participants throughout the study (<1.0 μg/mL). No skin irritation or other side effects were noted.

The binomial sign test was performed and demonstrated treatment with 6% topical gabapentin solution corresponded to a statistically significant (*p* = 0.03) improvement in median LPPAI scores from baseline to the final treatment visit (Figure 1). The clinical improvements of LPPAI scores were corroborated by data from patient questionnaires, where all participants had stable or improved scores on the dermatology life quality index^6^ and visual analogue scores for scalp‐related itch and pain. Additionally, physician assessment noted new terminal hair growth on the scalp of participants, including continued growth starting at visit 2 for two with LPP, visit 3 for the one with CCCA, and visit 5 for the one with FFA.

**FIGURE 1:**
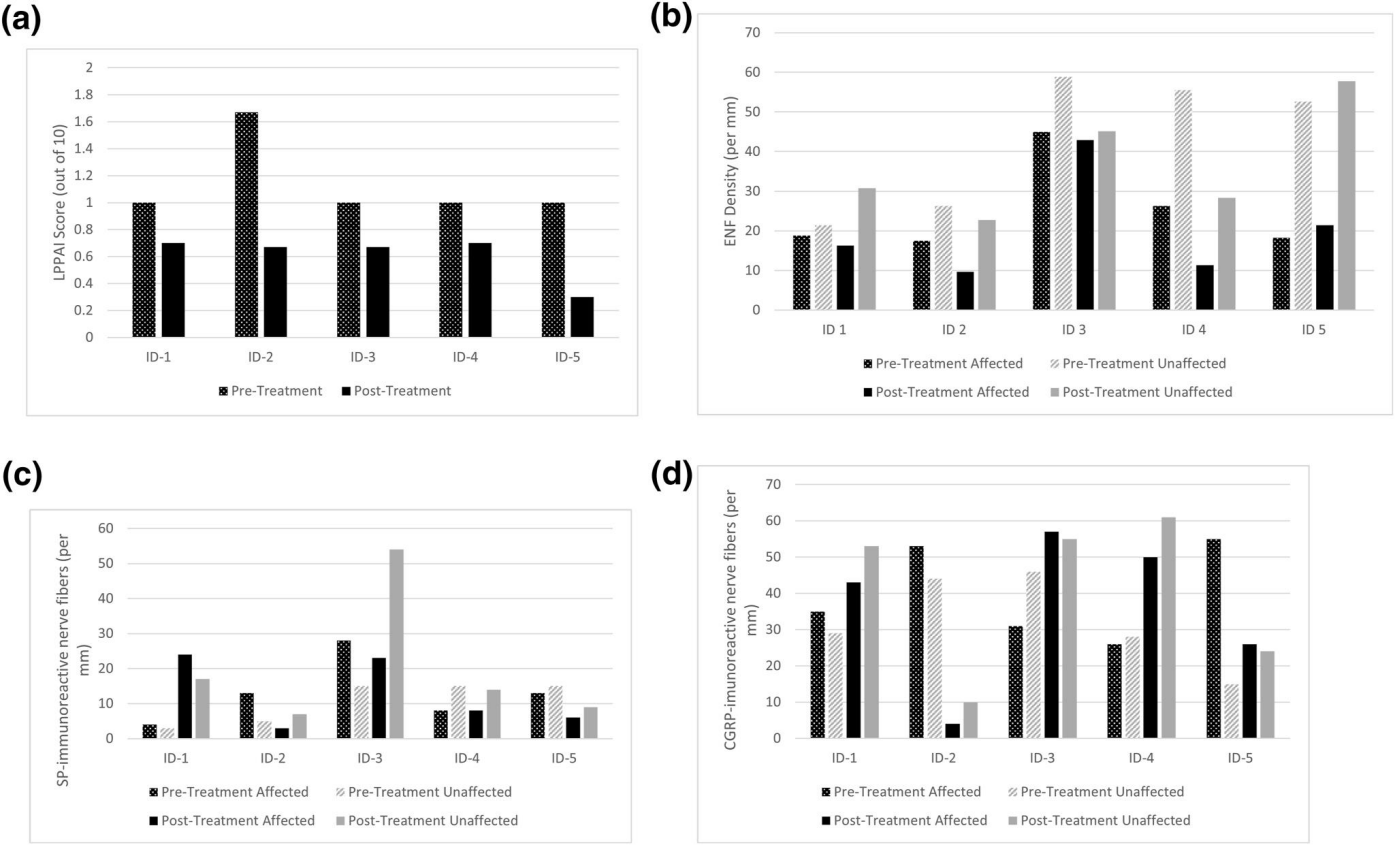
Graphs illustrating the change in data before and after treatment with 6% topical gabapentin solution for each participant. (a) All 5 participants demonstrated improved Lichen Planopilaris Activity Index (LPPAI) scores from visit 1 (1.13 ± 0.13 (SE)) to visit 5 (0.61 ± 0.08 (SE)), including two participants with 0.3‐point, one with 0.33‐point, one with 0.7‐point, and one with 1.0‐point reductions in scores. (b) Epidermal nerve fibre (ENF) density (per mm) was generally lower in affected scalp (23.1 ± 13.5(SD)) compared to unaffected scalp (39.7 ± 15.9(SD)), which is consistent with the definition of neuropathy. There were no clear group trends for affected scalp from visit 1 (25.5 ± 14.0(SD)) to visit 5 (20.7 ± 12.8(SD)) or for unaffected scalp from visit 1 (42.1 ± 17.4 (SD)) to visit 5 (37.4 ± 13.8 (SD)). (c) There were no clear group trends for substance P (SP) immunoreactive nerve fibre quantification (per mm) for affected scalp from visit 1 (13.2 ± 9.1 (SD)) to visit 5 (12.8 ± 9.9(SD)) or for unaffected scalp from visit 1 (10.6 ± 6.1(SD)) to visit 5 (20.2 ± 19.3(SD)). For affected scalp, three participants had decreased, one had relatively stable, and one had increased expression of SP after treatment. The only participant with FFA had notably elevated nerve fibre expression of SP in the post‐treatment unaffected scalp, but additional studies on other FFA participants are needed to confirm this finding. (d) There were no clear group trends for calcitonin gene‐related peptide (CGRP) immunoreactive nerve fibre quantification (per mm) for affected scalp from visit 1 (40.0 ± 13.2 (SD)) to visit 5 (36.0 ± 21.3(SD)) or for unaffected scalp from visit 1 (32.4 ± 12.8(SD)) to visit 5 (40.6 ± 22.3(SD)). For affected scalp, two participants had decreased and three had increased CGRP expression after treatment. FFA, frontal fibrosing alopecia.

ENFD was generally lower in affected scalp compared to unaffected scalp (Figure 1), suggesting a scalp skin neuropathy as defined by Kennedy et al.^7^ This is consistent with previous work on neurogenic inflammation in PCA.^5^ There were no clear trends for ENFD or SP and CGRP expression in relation to topical gabapentin treatment (Figure 1). Studies on systemic gabapentin suggest that it functions by inhibiting the alpha‐2‐delta subunit of voltage‐dependent calcium channels in the central nervous system,^1^ improving neuropathic symptoms by decreasing neuronal excitability.^8^ However, the pharmacodynamic actions of topical gabapentin are poorly characterised,^9^ and future research is needed to uncover the mechanisms underlying the clinical effects of topical gabapentin.

In summary, we provided evidence in this pilot study that monotherapy with 6% topical gabapentin solution was associated with improvement of lymphocytic PCA symptoms, LPPAI scores, and in some cases, physician documented hair growth. A future randomized controlled trial with large sample size should be conducted to confirm these findings and to further investigate cutaneous innervation and neuropeptide expression in a larger cohort. Our study only included participants with LPP, CCCA, and FFA, so it has limited generalisability to neutrophilic PCA diseases, such as folliculitis decalvans or dissecting cellulitis. Future basic and clinical research is needed to better understand the role of neurogenic inflammation in the PCAs. In the interim, topical 6% gabapentin may be an option for symptomatic lymphocytic PCA patients who are unresponsive to standard medical management.

## CONFLICT OF INTEREST STATEMENT

The authors declare no conflicts of interest.

## AUTHOR CONTRIBUTIONS

**Sara Bohjanen**: Formal analysis (lead); writing—original draft (equal). **Brian D. McAdams**: Investigation (equal); supervision (equal); writing—review and editing (equal). **Nasia Mead**: Investigation (equal). **Adam Loavenbruck**: Conceptualization (equal); supervision (equal); writing—review and editing (equal). **George L. Wilcox**: Conceptualization (equal); supervision (equal); writing—review and editing (equal). **Briana Paiewonsky**: Project administration (lead); writing—review and editing (equal). **Javed Shaik**: Writing—review and editing (supporting). **Maria K. Hordinsky**: Conceptualization (equal); investigation (equal); supervision (equal); writing—original draft (equal).

## FUNDING INFORMATION

Department of Dermatology, University of Minnesota; Scarring Alopecia Foundation

## ETHICS STATEMENT

The study received the University of Minnesota Institutional Review Board approval (1308M40801), and participants provided written, informed consent prior to enrolment.

## Data Availability

The data that support the findings of this study are available from the corresponding author upon reasonable request.
